# Optimal scaling of protein-water interactions coupled with targeted torsional refinements yields balanced force fields suitable for simulations of single-chain folded proteins, disordered polypeptides, and protein-protein complexes

**DOI:** 10.21203/rs.3.rs-5932820/v1

**Published:** 2025-02-27

**Authors:** Jeetain Mittal, Tien Phan, Priyesh Mohanty

**Affiliations:** Texas A&M University; Texas A&M University; Texas A&M University

## Abstract

All-atom molecular dynamics (MD) simulations based on physics-based force fields, serve as an essential complement to experiments for investigating protein structure, dynamics, and interactions. Despite significant advances in force field development, achieving a consistent balance of molecular interactions that stabilize folded proteins and protein-protein complexes while simultaneously capturing the conformational dynamics of intrinsically disordered polypeptides (IDPs), remains challenging. In this work, we systematically evaluated two current state-of-the-art force fields (i) AMBER ff03ws, and (ii) AMBER ff99SBws, by comprehensively assessing their performance on both folded domains and IDPs. By selectively scaling side chain-water interactions for uncharged residues, the refined AMBER ff03w-sc force field demonstrated improved conformational stability of folded proteins while maintaining accurate representations of IDPs. However, AMBER ff03w-sc failed to correct the discrepancies in NMR-derived ps-ns timescale backbone dynamics associated with flexible loops. Interestingly, AMBER ff99SBws retained its structural stability despite the application of upscaled interactions with water for both sidechain and backbone atoms and displayed robust agreement with NMR-derived backbone dynamics. Further, a targeted refinement of glutamine backbone torsion parameters, yielded AMBER ff99SBws-STQ′, which effectively resolved discrepancies associated with glutamine α-helicity predictions. Extensive validation against small angle X-ray scattering (SAXS) and NMR chemical shifts, revealed that both refined force fields accurately reproduced chain dimensions and secondary structure propensities of disordered peptides and prion-like domains. Importantly, both force fields reliably maintained the stability of protein-protein complexes over microsecond timescales. Our systematic refinement strategies provide improved accuracy and transferability for simulating diverse protein systems, from folded domains to IDPs and protein complexes.

## Introduction

Atomistic molecular dynamics (MD) simulations based on physics-based energy functions referred to as “force fields”, are now an invaluable and complementary tool to experiments in the investigation of protein structure, dynamics, and interactions associated with cellular processes.^1,2^ With significant advances in simulation hardware and the widespread availability of conformational sampling algorithms, accessing functionally relevant biomolecular dynamics and interactions which occur on the multi-microsecond to sub-millisecond timescale, are now routinely feasible.^3^ Importantly, these advances have also enabled a thorough investigation of the inaccuracies associated with protein force fields in comparison to experiment with regard to their propensity to populate various secondary structure classes and the strength of non-bonded interactions between various chemical groups.^4^

After nearly two decades of force field parameterization against crystallography, spectroscopic and ab initio calculations, modern force fields belonging to the four major families - AMBER, CHARMM, OPLS and GROMOS, succeeded in providing a reasonable description of the structure and dynamics of folded proteins but performed poorly in their description of structural ensembles for short peptides in solution.^5,6^ At present, a major emphasis and goal of modern atomistic force field development is the parameterization of transferable models which can simultaneously describe the structural stability of folded domains while capturing the transient secondary structure and global dimensions of intrinsically disordered proteins (IDPs).^7–11^ A major step towards arriving at these goals involved the extensive comparison of simulation ensembles against solution NMR observables (chemical shifts and J-couplings) for weakly structured peptides, first carried out by Best and Hummer.^12^ Based on these observations, global empirical corrections applied to either ϕ or ψ backbone torsional potential for nonglycine/proline residues to adjust the helix-coil or PPII-β conformational equilibria which led to amber ff99SB*/ff03*/ff99SBϕ^13,14^ and charmm22*/36^15,16^ force fields which achieved an improved balance between the secondary structure classes and successfully folded both α-helical and β-sheet proteins. Notably, a similar strategy based on comparison with NMR J-couplings was also utilized in improving the description of sidechain rotamer configurations for isoleucine (I), leucine (L) and aspartate (D) residues in ff99SB, giving rise to ff99SB*-ILDN.^5,15^

Despite extensive reparameterization of backbone and sidechain torsion potentials, the widespread use of primitive three-site water models such as TIP3P, consistently led to weak temperature-dependent cooperativity for protein folding,^17^ overly collapsed structural ensembles for IDPs^6,18,19^ and excessive protein-protein association across the major force field families.^20^ To remedy issues arising due to weak protein-water interactions, subsequent efforts were devoted towards the adoption and/or development of accurate four-site (rigid) water models^21–24^ which aimed to increase the strength of protein-water interactions. Among these efforts, the amber ff99SB*/ff03* force fields were combined with the TIP4P2005 water model, along with scaled protein-water interactions and a readjustment to the global correction for ψ backbone dihedrals (for non-Gly/Pro residues) applied to maintain the correct helix-coil equilibrium for model peptides.^25^ These modifications resulted in the ff99SBws/ff03ws force fields which showed considerable improvements in global and structural properties computed for disordered polypeptides (from smFRET and SAXS) and reduced excessive protein-protein association.^25–27^ These efforts were soon followed by a reparameterization of charmm36 (charmm36m) to alleviate its tendency to form left-handed α-helices, including the adoption of a modified TIP3P water with additional LJ parameters on its hydrogen atoms to enhance protein-water interactions and improve the conformational description of IDPs.^28^ Robustelli et al. paired ff99SB-ILDN with a modified TIP4P-D water model and a Lennard-Jones parameter modification to the increase the strength of backbone hydrogen bonding, resulting in ff99SB-disp force field which showed state-of-the-performance in the description of both folded and disordered protein ensembles across a wide range of test systems chosen in the study.^29^ Along similar lines, a version of ff99SB optimized to reproduce the residue-specific torsional angle distributions observed for the PDB coil library, RSFF2,^30^ yielded highly accurate thermodynamics of protein folding when coupled to TIP4P-D water (RSFF2+).^31^ The most recent reparameterization of the official amber force field - ff19SB,^32^ was also recommended to be paired with the four-site OPC water model as it gives rises to more accurate IDP conformational ensembles with respect to experiment.^33^ As an alternative to the adoption of computationally expensive four-site water models for protein simulations, Yoo and Aksementiev performed a reparameterization of atom pair-specific Lennard-Jones parameters in charmm/amber force fields against osmotic pressure data to mitigate the overstabilization of salt bridges and hydrophobic interactions in TIP3P water.^34–36^

Despite steady progress towards the development of “balanced” force fields over the last decade, numerous independent investigations reported discrepancies pertaining to certain IDP sequences, folded protein stability, and protein-protein association. For instance, the ff03ws force field significantly overestimated (> 16%) the chain dimensions (Rg/R_h_) of the RS peptide compared to experiment.^18^ Further, it was observed in replica exchange simulations of WW domain domains that ff03ws strongly destabilized the folded state.^35^ Two independent studies which investigated the suitability of state-of the-art force fields such as charmm36m, ff19SB and ff99SB-disp revealed discrepancies in all three force fields regarding the solubility of the aggregation-prone Aβ_16−22_ peptides^37^ and ubiquitin self-association.^38^ These studies revealed that ff99SB-disp overestimates the strength of protein-water interactions, failing to predict the formation of β-aggregates for Aβ_16−22_ and weak dimerization (millimolar affinity) of ubiquitin. In contrast, charmm36m correctly predicted the aggregation propensity of Aβ_16−22_ wild type and two mutants while ubiquitin self-association was found to be too strong. ff19SB-OPC exhibited intermediate behavior, giving rise to only a small population of β-aggregates for Aβ16–22 but successfully predicted weak dimerization of ubiquitin through the known interacting surfaces. To address issues related to weak protein-protein association for ff99SB-disp, Piana et al. recently proposed a reparameterization of both dihedral parameters and non-bonded interactions against osmotic pressure data.^39^ While the reparametrized force field, referred to as DES-amber, increased the stability of protein complexes, the association free energies were still underestimated compared to experiment for some systems. Collectively, the above findings indicate that the optimal balance between protein-protein and protein-solvent interactions among modern classical force fields is yet to be fully realized.

Given the above-mentioned issues with current state-of-the-art force fields, in this study, we have carefully re-evaluated the accuracy and transferability of two such force fields, namely amber ff03ws and ff99SBws,^25^ in describing the structural dynamics of both folded and disordered domains. For ff03ws, we observed that a simple modification to protein-water scaling scheme, i.e., selective scaling of sidechain-water interactions for neutral and polar residues only (and not all atoms as performed previously) led to a remarkable improvement in the stability of various folded proteins while simultaneously yielding structural ensembles for short peptides (RS, histatin, S(129–146)) and prion-like IDPs (TDP-43, FUS) that are in excellent agreement with experiment. The modified scaling scheme, however, only led to modest improvements in the ps-ns timescale backbone dynamics (order parameter - S^2^) of folded domains which were previously studied by NMR. In contrast, ff99SBws maintained the stability of folded domains and yielded S^2^ values which were already in excellent agreement with NMR without the need to readjust the original scaling scheme for protein-water interactions. Further, ff99SBws also yielded structural ensembles of RS peptide and Histatin which are in excellent agreement with experiment. A discrepancy was observed for Huntingtin Exon1 wherein the helicity of the polyglutamine tract was overestimated compared to NMR. Here, the incorporation of a minor modification to backbone dihedral angle of glutamine (Q) led to a helicity profile which was in good agreement with experiments.

In conclusion, we propose two balanced force fields, namely ff03w-sc and ff99SBws-STQ′, which are expected to be well-suited for simulating a wide range of protein systems encompassing folded, intrinsically disordered proteins and protein-protein complexes.

## Results

### Stability of folded proteins.

Recent efforts to develop “balanced” force fields–achieved by modifying protein-water van der Waals (vdW) interactions or re-parameterizing water models to incorporate stronger dispersion forces–have significantly improved the prediction of global properties for many intrinsically disordered proteins (IDPs), while maintaining essential local structural features.^9,23,25,26,29^ However, these advancements come with a potential drawback: the strengthened protein-water interactions to better represent the denatured state ensemble may inadvertently compromise the conformational stability of folded proteins and peptides.^23,25,29,40^ To evaluate this potential issue with amber ff03ws and ff99SBws force fields, we investigated the stability of two model systems: Ubiquitin (PDB ID: 1D3Z),^41^ a highly conserved protein known for its millisecond-scale stability,^42,43^ and the Nle-double mutant of the Villin headpiece (HP35) (PDB ID: 2F4K),^44^ an ultrafast-folding protein.^44,45^

**Figure 1:**
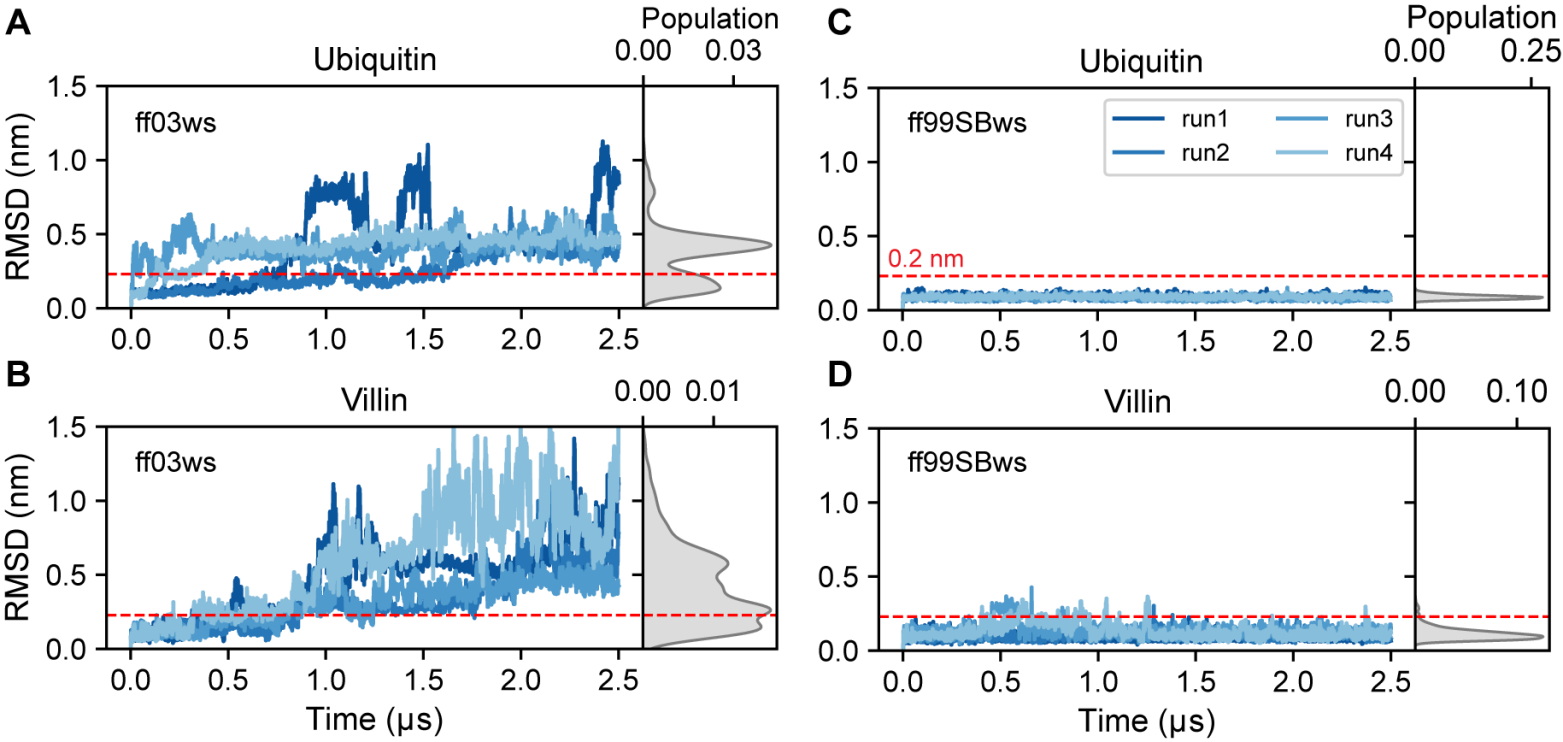
Stability of folded proteins. Analysis of backbone-based RMSD (Root Mean Square Deviation) for ubiquitin (**A, B**) and Villin head peace (**C, D**) simulated with the amber ff03ws and ff99SBws force fields, respectively, across four independent simulations. The red dashed line indicates an RMSD of 0.2 nm, serving as a reference threshold for stability.

Four independent simulations (2.5 μs each) performed using ff03ws revealed significant instability in both proteins. For Ubiquitin, the most populated state deviated by approximately 0.4 nm in backbone RMSD (excluding the flexible C-terminal tail, residues 71–76) from the X-ray crystal structure, accompanied by substantial local unfolding of the α-helix (Figs. 1A, S1A Movie S1). Similarly, simulations of Villin HP35 exhibited pronounced structural deviations from the native state and unfolding events after 1 μs (Figs. 1B, S1B Movie S1). In contrast, the ff99SBws force field effectively maintained the structural integrity of both Ubiquitin and Villin HP35 over the microsecond timescales. For Ubiquitin, RMSD and RMSF values remained consistently low (< 0.2 nm) across all four independent simulations (Figs. 1C, S1A, Movie S2). Villin H35 exhibited slightly larger structural fluctuations, with minor conformational rearrangements in the C-terminus (residues 30–35); however, the overall RMSD remained predominantly below 0.2 nm (Fig. 1D, S1B, Movie S2). Our comparative analysis reveals that ff99SBws force field effectively maintains native conformational states of both Ubiquitin and Villin HP35, whereas ff03ws exhibits notable destabilization with significant conformational rearrangements, indicating its potential limitations for extended MD simulations of folded proteins.

### Modifications to the protein-water scaling scheme in amber ff03ws.

**Figure 2:**
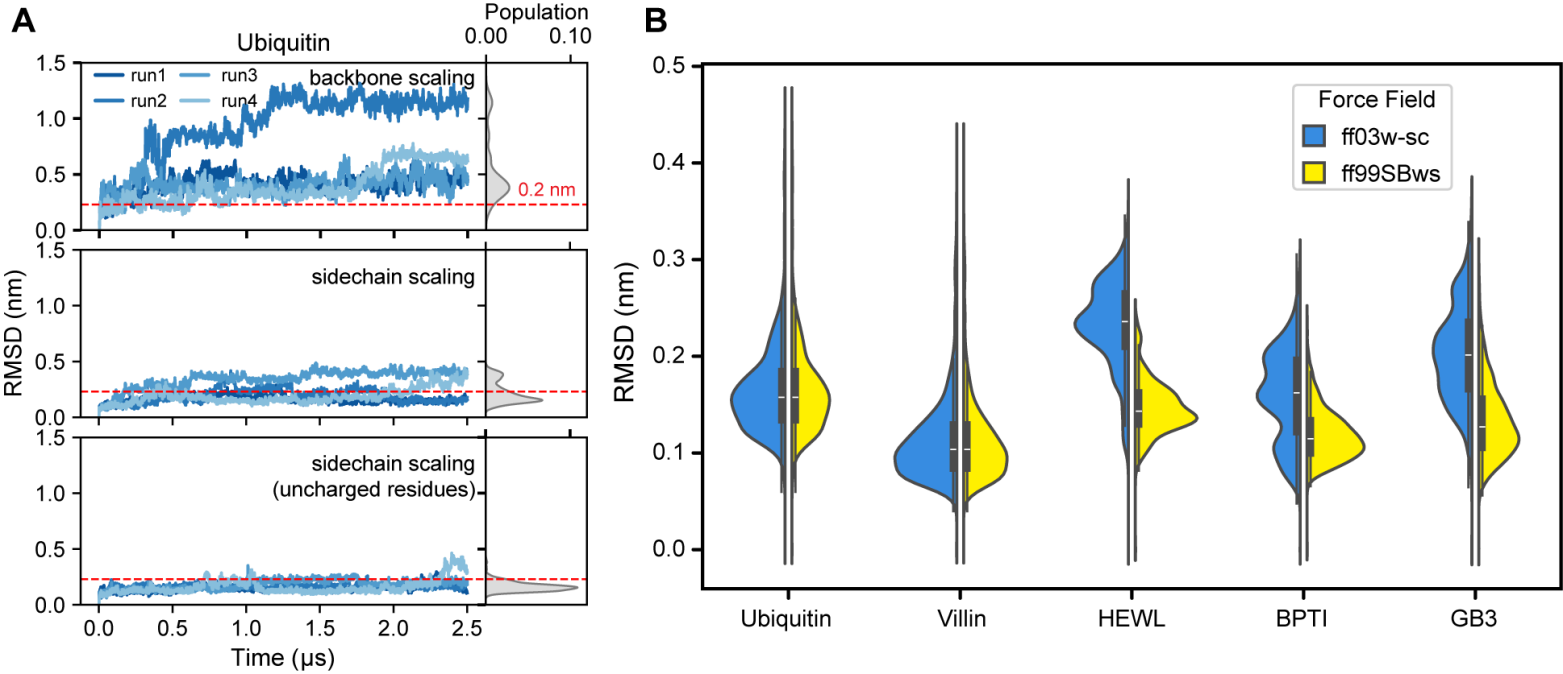
Enhanced folded protein stability by optimizing scaling conditions derived from amber ff03ws. (A) Backbone-based RMSD of ubiquitin with applied backbone scaling, sidechain scaling, and sidechain scaling (excluding charged residues), respectively. The red dashed line indicates an RMSD of 0.3 nm. (B) Population distribution of RMSD for Ubiquitin, Villin, GB3, BPTI, and HEWL proteins simulated in ff99SBws and ff03ws with sidechain scaling (excluding charged residues) applied.

In folded proteins, structural stability is maintained through a combination of hydrogen bonding involving primarily the polypeptide backbone, and hydrophobic/electrostatic interactions mediated by sidechain atoms. To investigate the relative contributions of the backbone and side chains to Ubiquitin’s stability, we tested two new variants of the ff03ws force field. These variants selectively scaled protein-water Lennard-Jones (LJ) interactions by 10% (as described by Best et al.^25^) for either backbone heavy atoms (backbone scaling) or side chain atoms (side-chain scaling, excluding glycine). Backbone scaling resulted in increased structural deviations (Figs. 2A, S2C Movie S3), indicating reduced stability and a significant impact on protein dynamics. In contrast, side-chain scaling significantly improved overall structural integrity relative to the native state, albeit with persistent RMSD fluctuations exceeding 0.2 nm (Figs. 2A, S2C Movie S3).

Surface-exposed charged residues are crucial to protein stability as they interact strongly with the solvent and maintain essential electrostatic balances.^46^ We strategically excluded these residues from our side-chain scaling approach for several reasons. First, the parent ff03 already demonstrates robust performance in modeling salt bridge interactions, showing consistent equilibrium association constants and binding probabilities irrespective of the water model.^47^ Second, the ff03w force field, derived from ff03* paired with the optimized TIP4P/2005 water model, yields charged side-chain hydration free energies that closely approximate experimental measurements.^48^ Third, within our side-chain scaling framework, charged residues–which comprise approximately 30% of ubiquitin–exhibited notable deviations in solvent-accessible surface area (SASA) relative to the experimental structure (Figures S2A, B, D). Excluding charged residues from the side-chain scaling scheme led to substantially enhanced ubiquitin stability, characterized by RMSD values consistently below 0.2 nm and reduced the solvent accessibility of charged residues (Figs. 2A, S2C, D, Movie S3). These improvements indicate a more stable folded conformation.

To assess the robustness of this modified force field, designated ff03w-sc, we applied it to the folded state of Villin HP35, which had previously unfolded in simulations using the original ff03ws. With ff03w-sc, Villin HP35 exhibited substantially reduced structural fluctuations, with backbone RMSD and RMSF values consistently remaining below 0.2 nm (Figure S3A, Movie S4), indicating improved structural stability. We further tested ff03w-sc on three additional folded proteins–GB3 (PDP ID: 1P7E),^49^ BPTI (PDB ID: 5PTI),^50^ and HEWL (PDB ID: 6LYZ)^51^–known to destabilize in ff03ws simulations.^29^ While the backbone RMSD distributions for these proteins were relatively broad, they remained centered around 0.2 nm (Fig. 2B), suggesting a moderate degree of flexibility, primarily localized to loop regions (Figures S3B-D). Overall, the proteins retained native-like conformations throughout μs-long simulations.

Interestingly, simulations of all five folded proteins using the ff99SBws force field consistently exhibited lower RMSD values (< 0.2 nm) and narrower distributions (Figs. 2B, S3B-D), indicating greater stability and closer adherence to native structures. Notably, ff99SBws also employs a 10% increase in protein-water interaction strength for all protein atoms–similar to the approach used in the original ff03ws.^25^ These findings demonstrate that ff03w-sc improves protein structural integrity over microsecond timescales, while underscoring the superior performance of the original ff99SBws in maintaining the structural stability of folded proteins without further modifications.

### Chain dimensions of IDPs.

**Figure 3:**
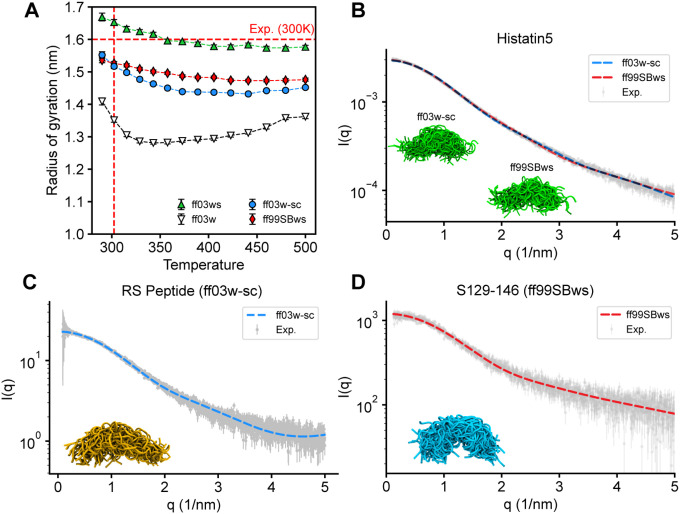
Chain dimensions of IDPs. **(A)** Radius of gyration of CspM34 as a function of temperature. Results are shown for ff03ws (green), ff03w-sc (blue), ff99SBws (yellow), and ff03w (black), respectively. The experimental value at 300 K is indicated by the red dashed line. **(B-D)** SAXS profiles of Histatin5, RS Peptide, and S(129–146) peptide. The experimental data (gray), taken from Saga et al.^56^ for Histatin5, Rauscher et al.^18^ for RS peptide, and Koren et al.^57^ for S(129–146), are overlaid with the simulated scattering profiles. The structure inset displays the conformational ensembles.

To evaluate the suitability of the force fields in simulating IDPs, we determined the temperature-dependent radius of gyration (R_g_) for a 34-residue fragment of Cold-shock protein (CspM34) using parallel tempering in a well-tempered ensemble (PT-WTE) to enhance conformational sampling.^52,53^ Fig. 3A presents the R_g_ of CspM34 as a function of temperature for ff03w-sc and ff99SBws, compared to two reference force fields, ff03w and ff03ws. An experimental R_g_ estimate for CspM34 at 300 K, derived from FRET measurements using a Gaussian chain model,^54,55^ is approximately 1.6 nm. Across the temperature range, ff03ws consistently maintained extended conformations, with an R_g_ value of 1.65 ± 0.01 nm at 300K. In contrast, ff03w produced more compact structures, with an R_g_ of 1.35 ± 0.1 nm at 300K and decreasing further at elevated temperatures. Both ff03w-sc and ff99SBws yielded intermediate chain dimensions, with R_g_ values of 1.52 ± 0.01 nm and 1.53 ± 0.01 nm at 300 K, respectively.

We further investigated the influence of side-chain scaling on the conformational dimensions of CspM34. Side-chain scaling variants, both with and without charged residues, produced comparable R_g_ values (1.51 ± 0.01 nm and 1.52 ± 0.01 nm at 300K, respectively), although excluding charged residues led to a slightly more collapsed conformations at higher temperatures (Figure S4A). These results suggest that the side-chain scaling of uncharged residues does not substantially alter the chain dimensions of CspM34. We note that a 10% increase in protein side chain-water interactions used in ff03w-sc represents a near-optimal value for balancing the structural integrity of folded proteins and preventing over-collapse of disordered proteins. While increasing protein side chain-water affinity can achieve more realistic chain dimensions for disordered proteins, it potentially destabilizes folded proteins (Figure S4A-C). Notably, introducing only side-chain scaling to ff99SBws resulted in more collapsed conformations of CspM34 (Figure S4D).

To assess the accuracy of chain dimensions predicted by ff03w-sc and ff99SBws, we compared their small-angle X-ray scattering (SAXS) profiles to experimental data. As an initial validation system, we selected Histatin5, a 25-residue peptide enriched in hydrophilic amino acids (Table S1), which are known to promote conformational disorder compared to hydrophobic residues that are typically found in stable protein cores. Using PT-WTE simulations, we computed the SAXS profiles from the 302 K replica. Figure 3B illustrates the comparison between experimental and simulated SAXS scattering curves for Histatin5. Both force fields demonstrated excellent agreement with experimental data, within error margins. Notably, the R_g_ values derived from the experimental SAXS profile^56^ (13.9 ± 0.1 Å) closely matched the predictions from both ff03w-sc and ff99SBws ensembles (13.8 Å).

Further validation was performed using the RS-peptide and the fragment S(129–146) of the NFLt sequence (Table S1). Previous studies reported that ff03ws overestimated the R_g_ of RS-peptide by approximately 16%.^18^ In contrast, ff03w-sc produced the SAXS profiles in excellent agreement with experimental measurements (Fig. 3C), yielding a theoretical R_g_ value of 12.7 ± 0.1 Å compared to the experimental value of 12.6 ± 0.1 Å. For the S(129–146) fragment,^57^ which features alternating charged blocks that potentially facilitate salt-bridge interactions, the ff99SBws ensemble predicted a SAXS profile that aligned well with experimental data (Fig. 3D). The predicted R_g_ value of 12.8 ± 0.1 Å agrees closely with the experimental measurement of 12.9 ± 0.1 Å. Although ff99SB-based force field has been previously reported to overestimate salt-bridge strength,^47^ this effect appears negligible in small IDPs, presumably due to their inherent conformational flexibility and dynamic nature.

Collectively, these results demonstrate that both ff03w-sc and ff99SBws achieve robust agreement with experimental R_g_ values and SAXS profiles, thereby establishing their reliability in modeling the chain dimensions of small IDPs.

### Secondary structure predictions for IDPs.

**Figure 4:**
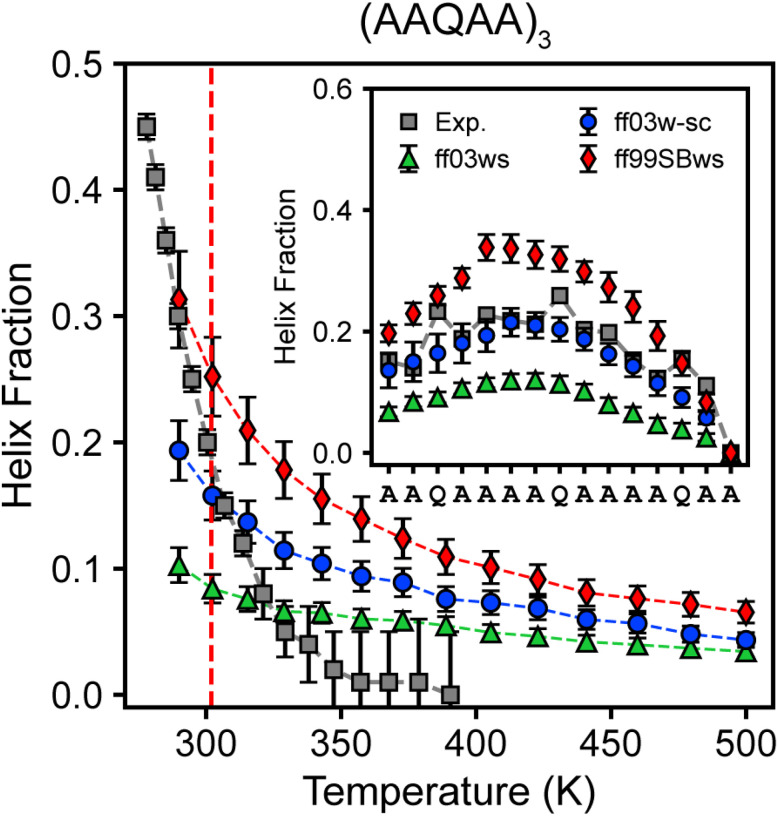
Temperature dependence of structural properties of (AAQAA)_3_ model peptide. Helix fraction of (AAQAA)_3_ as a function of temperature simulated in ff03ws, ff03w-sc, and ff99SBws, respectively. The inset shows the residual helix fraction at 300K. Error bars indicate the standard deviation.

While accurately sampling chain dimensions in disordered peptides is crucial, it is equally important to ensure that modifications to protein-water interactions do not compromise the stability of folded motifs, such as helices, which may be transiently populated in unfolded states and IDPs such as the TDP-43 low complexity domain.^58^ To assess this balance, we investigated a 15-residue helix-forming peptide Ace-(AAQAA)_3_-NH_2_, which exhibits approximately 30% helix content at 300 K and has a well-characterized temperature-dependent helix propensity from previous NMR studies and has become an established reference system for force field validation.^59^ Fig. 3 illustrates the temperature-dependent helix propensity computed using ff03ws, ff03w-sc, and ff99SBws force fields. ff03w-sc, incorporating scaled side chain-water interactions, demonstrated enhanced helix stability compared to ff03ws and accurately reproduced a residual helix fraction closely aligned with NMR data at 300 K. In contrast, ff99SBws consistently overestimated helix content across the temperature range and predicted elevated helicity for most residues (Fig. 4, inset). The results highlight the significant improvement achieved by ff03w-sc in stabilizing helix formation of the (AAQAA)_3_ peptide, while emphasizing necessary refinements for ff99SBws to improve residual helicity predictions in disordered states.

**Figure 5:**
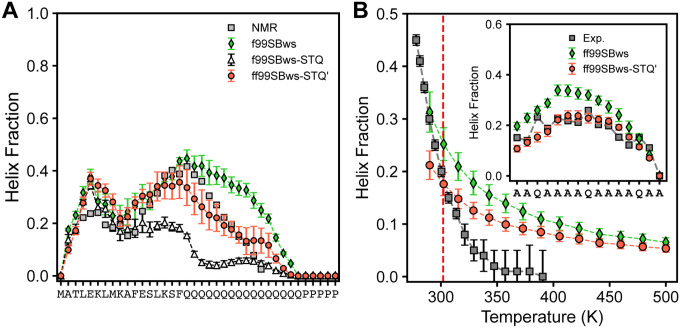
Fine-tuning torsion parameters of Glutamine in amber ff99SBws-STQ. **A.** Residual helix fraction of H16, simulated in three amber ff99SBws-based force fields: ff99SBws, ff99SBws-STQ, and ff99SBws-STQ¢ (k_y_ (Q) = 1.5 kJ/mol). NMR-derived results are taken from Urbanek et al.^61,62^ **B**. Residual helix fraction of (AAQAA)_3_ as a function simulated in ff99SBws and ff99SBws-STQ¢, respectively. The inset shows the temperature-dependent helix fraction. Error bars indicate the standard deviation.

Recognizing the limitations of existing force fields, particularly their inaccuracies in capturing amino acid-specific conformational preferences across diverse sequence contexts, we implemented targeted refinement to address these challenges. Our initial modification led to the development of ff99SBws-STQ, which reduced the bias toward helical structures by adjusting the backbone torsion potential parameter (k_ψ_) for serine (S), threonine (T), and glutamine (Q) from 2.0 kJ/mol to 1.0 kJ/mol.^60^ This refinement substantially improved all-atom simulations of low-complexity domains (LCDs) of TAR DNA-binding protein 43 (TDP-43), Fused in Sarcoma (FUS), and the C-terminal heptad repeat domain of RNA Polymerase II (RNA Pol II), particularly in predicting ^13^C NMR chemical shifts and secondary structure propensities. However, simulations of the N-terminal fragment of Httex1, comprising N17 (17 amino acids), Q16 (polyglutamine), and a minimal stretch of five prolines (P5), revealed persistent discrepancies in residual helicity predictions in the polyQ region: ff99SBws exhibited substantial overestimation, while ff99SBws-STQ showed significant underestimation (Fig. 5A). To address this issue, we revisited the torsion correction for glutamine and tested intermediate k_ψ_ values (between 1.0 kJ/mol and 2.0 kJ/mol). We found that k_ψ_ = 1.50 kJ/mol effectively balanced the bias, accurately reproducing experimental residual helicity in the polyQ region, with an RMSD of 5.1% between simulated and experimental helix fractions (Table S2). The revised force field, designated ff99SBws-STQ′, not only resolved the residual helicity discrepancies in the polyQ region but also demonstrated improved accuracy in reproducing experimental helix fraction of the (AAQAA)_3_ peptide across a wide temperature range, achieving excellent agreement with NMR measurements at 300 K (Fig. 5B).

**Figure 6:**
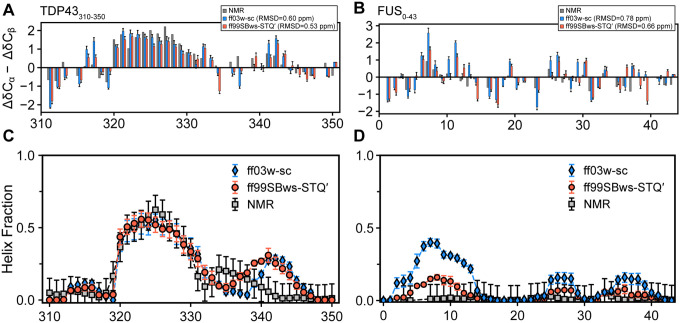
Secondary structure prediction for TDP43 and FUS fragments simulated in AMBER ff03w-sc and ff99SBws-STQ¢. (**A, B**) Chemical shift deviations (Δ*δ*C_α_ − Δ*δ*C_β_) for TDP43_310–350_ and FUS_0–43_ as a function of residue index, respectively. (**C, D**) Residual helix fraction of TDP4_3310–350_ and FUS_0–43_, respectively. Error bars for DSSP indicate the standard deviations between 10 ns periods in simulation trajectories. Error bars for NMR-derived *δ*2D population per residue are 0.1 (10%).

To further evaluate the applicability of ff03w-sc and ff99SBws-STQ′, we examined prion-like LCDs, including FUS_0 − 43_, FUS_120 − 163_, TDP-43_310−350_, and RNA Pol II_1927 − 1970_^63–65^. We strategically selected 44-residue fragments of these proteins to optimize computational efficiency while maintaining biological relevance and employed PT-WTE simulations to achieve converged equilibrium properties. All subsequent analyses were performed using the replica at 302 K. Conformational preferences were quantitatively assessed through secondary chemical shift difference (ΔδC_α_ – ΔδC_β_), a metric that discriminates between helical (positive values) and β-sheet (negative values) regions by comparing observed C_α_ and C_β_ chemical shifts against random coil reference values. Figure 6A shows excellent agreement between simulated and experimental data in the helix-forming region (residue 321–330) in TDP-43310–350, an alanine-rich segment within the LCD. Both force fields predicted the correct helical propensities across the chosen LCD fragments with the exception of the N-terminal region (residue 6–13) in FUS_0 − 43_ which was overly helical (Figs. 6), a discrepancy also reported for other force fields.^60^ The RMSDs of simulated chemical shifts relative to experimental measurements for both force fields were less than the prediction error of SPARTA+ (± 1 ppm for carbon chemical shifts^66^), highlighting their reasonable accuracy in modelling IDP conformational ensembles.

In addition, we compared secondary structure propensities calculated from simulation trajectories using DSSP^67^ to those predicted from experimental NMR chemical shifts using the δ2d web server.^68^ For TDP-43, both force fields showed excellent correlation with experimental observations, accurately capturing partial helicity in the alanine-rich segment. In contrast, FUS_0 − 43_ simulations revealed distinct behaviors: ff03w-sc exhibited elevated helical populations (~ 40%) in the N-terminal region (residue 6–13), while ff99SBws-STQ′ simulations predicted more transient helical conformations (< 15%), closely aligning with NMR observations (Figs. 5C, D, and S5). Analysis of additional polar-rich LCDs, including FUS_120 − 163_ and RNA Pol II_1927 − 1970_, demonstrated that both ff03w-sc and ff99SBws-STQ′ accurately predicted low helical populations (< 20%), consistent with experimental measurements (Figure S6). For reference, a comparative analysis between ff99SBws-STQ′ and ff99SB-disp (Figures S7) revealed that ff99SB-disp overestimated the helicity in the polyQ tract of Httex1 (residue 28–32) and the N-terminal region of FUS_0 − 43_ (residue 6–16), while underestimating helix propensity in the helix-forming region of TDP-43_310−350_ (residue 321–330).

### Protein-Protein complexes.

**Figure 7:**
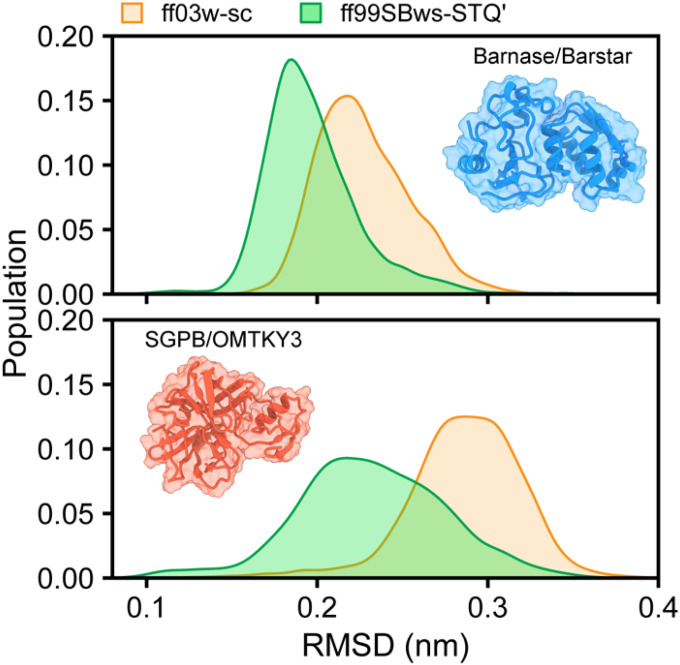
Protein complexes stability. Backbone-based RMSD distributions of two protein complexes Barnase/Barstar and SGPB/OMTKY3 simulations using ff03w-sc and ff99SBws-STQ¢ force fields. The insets present the experimental structures of the complexes.

Modeling protein-protein interactions within molecular complexes remains a significant challenge for most current force fields, as the multifaceted balance of water-protein, intra-protein, and water-water interactions has been primarily optimized for isolated or disordered proteins. Previous simulations using state-of-the-art force fields (e.g., ff99SB-disp, charmm36m, and RSFF2+) have reported instability and dissociation of many protein complexes on microsecond timescales.^39^ Therefore, we further examine the ability of ff03w-sc and ff99SBws-STQ to model protein-protein complexes, focusing on Barnase/Barstar (PDB ID: 1BRS)^69^ and SGPB/OMTKY3 (PDB ID: 3SGB)^70^ – two systems previously identified as challenging for maintaining stability of protein-protein interactions.^39^ For each complex, we conducted four independent 5-μs simulations initiated from the experimental structure. In both Barnase/Barstar and SGPB/OMTKY3, the backbone RMSD values of the overall complexes and individual proteins consistently remained stable throughout the simulations (Figs. 7 and S8, Movies S5, S6), demonstrating remarkable structural integrity of the complexes over microsecond timescales. These results highlight the capability of ff03w-sc and ff99SBws-STQ′ to reliably maintain the stability of protein-protein interaction networks in complex biomolecular assemblies.

## Discussion and Conclusion

In this study, we systematically re-evaluated two previously developed amber force fields - ff03ws and ff99SBws, to address their limitations in stabilizing folded proteins and accurately modeling IDPs. By selectively scaling sidechain-water interactions, the modified ff03w-sc force field significantly enhanced the structural stability of folded proteins, such as Ubiquitin and Villin HP35, which exhibited destabilization or partially unfolding with the original ff03ws. Notably, ff99SBws maintained native state stability without requiring additional rescaling. We further revisited ff99SBws-STQ force field, which incorporates residue-specific backbone torsional modifications to ff99SBws base model, previously validated for LCD conformational sampling. By fine-tuning the backbone torsional parameters for glutamine, the updated ff99SBws-STQ′ force field effectively mitigated over-predicted helicity in the polyQ tract of Huntingtin Exon1, bringing simulated residual helicity in line with experimental data. This targeted approach demonstrates the value of optimizing force field parameters to resolve system-specific inaccuracies while maintaining overall transferability. Comprehensive validation against experimental SAXS profiles and NMR data for diverse systems including Histatin5, RS-peptide, S(129–146) fragment, and prion-like domains - substantiates the robustness and reliability of the proposed refinements.

An additional critical test of the refined force fields involved evaluating their ability to maintain protein-protein complex stability, which has been recognized challenge for many existing models due to the delicate balance required between protein-water and protein-protein interactions. Using the Barnase/Barstar and SGPB/OMTKY3 complexes as model systems, we demonstrated that both ff03w-sc and ff99SBws-STQ′ reliably maintain native stability of these complexes over microsecond simulations. This robust performance stands in contrast with previous reports of instability and premature dissociation observed in similar complexes using other state-of-the-art force fields, such as ff99SB-disp and charmm36m.^39^ These findings substantially extend the applicability of the refined force fields to the investigation of biomolecular complexes, providing a reliable computational framework for studying protein-protein interfaces, molecular recognition mechanisms, and the hierarchical assembly of functional protein complexes.

A persistent challenge with ff03-based force fields is their tendency to overestimate backbone flexibility, particularly in disordered loop regions of folded proteins. While these force fields accurately reproduced the conformational rigidity of well-structured regions, such as α-helices and β-sheets (with S^2^ values > 0.8), significant deviations from experimental S^2^ values were observed in loop regions (Figure S9A). For example, simulations of Ubiquitin using ff03*/TIP3P maintained global structural stability, yet residues within loops, notably glycine residues (G47, G53) and charged residues (K48, R54, E64), exhibited greater flexibility relative to experimental S^2^ values (Figure S9A). Among ff03-based force fields paired with TIP4P/2005 water model, ff03w-sc achieved an improved balance between backbone rigidity and flexibility but still showed discrepancies in loop regions, highlighting the difficulty in accurately capturing flexible loop dynamics. The CMAP-optimized ff03CMAP force field represented another effort to address these imbalances, but it often favored either folded or disordered systems depending on the water model (e.g., TIP4P-Ew, TIP4P-D), rather than offering a unified solution.^71^ In contrast, ff99SB-based force fields - such as ff99SBw, ff99SBws, and ff99SB-disp–consistently exhibited higher order parameters and aligned more closely with experimental data, particularly in loop regions (Figure S9B), reflecting their ability to capture backbone dynamics across both folded and disordered regions.

The refinements introduced in this study have addressed many challenges, including the over-predicted helicity in specific regions of LCDs. Both force fields demonstrate consistently small secondary chemical shift deviations Δ(ΔC_α_ - ΔC_β_) (< 1 ppm) across many residue types (Figure S10), highlighting their improved accuracy. However, some discrepancies remain. For example, significant deviations in secondary chemical shifts persist for polar residues, notably Ser and Thr, in ff03w-sc. While both ff03w-sc and ff99SBws-STQ′ effectively captured secondary structure propensity for several polar-rich sequences (TDP-43, FUS, and RNA Pol II fragments), the helicity in the N-terminal region of FUS_0 – 43_ remained overestimated relative to experimental data. These observations indicate persistent sequence-specific biases, particularly in polar residue clustering and repetitive sequence motifs, necessitating further parametric optimization. Given the sequence diversity in LCDs and IDPs, comprehensive testing across a wider range of sequence types–including hydrophobic-rich, charged, and mixed-composition domains–will be essential for evaluating and improving the performance of these force fields.

Choosing an appropriate force field for MD simulations require careful consideration for specific systems, as no single force field is universally optimal. A preliminary literature review of studies involving similar systems is essential for identifying force fields with recognized strengths and weaknesses. For example, ff99SBws-STQ′ has emerged as a robust choice for simulating both folded proteins and IDPs due to its effective balance of rigidity and flexibility. This force field demonstrates strong agreement with experimental observations of full-length multidomain proteins such as HP1α and TPD43,^72–75^ reliably reproduces the experimental ps-ns dynamics, as indicated by its accurate modeling of spin relaxation data (e.g., R_1_, R_2_, and hetNOE), reflecting the disordered nature of the EWS LCD and its regional variations in relaxation behavior.^76^ Although ff03ws tends to overestimate backbone flexibility in folded protein loops, it demonstrates strong agreement with R_1_, R_2_, and hetNOE data for disordered and partially disordered proteins, effectively modeling extended conformations and rotational dynamics under experimental ionic strengths.^77^ For systems characterized by a high density of charged residues–such as DNA-binding proteins, their complexes, or highly charged IDPs – ff03w-sc offers advantages, as it effectively mitigates the over-stabilization of salt bridges, a common artifact observed in many force fields.^47^

In conclusion, this study advances the ongoing efforts to refine protein force fields, facilitating their utility in biomolecular simulations. The proposed force fields– ff03w-sc and ff99SBws-STQ′–achieve a balanced description of both folded and IDP ensembles, offering improved accuracy and reliability for studying diverse protein systems under physiological and pathological conditions. These advancements establish a robust and versatile computational framework for simulating complex biomolecular processes, narrowing the gap between theoretical predictions and experimental observations. Future efforts will extend these improvements to larger, multi-domain proteins and explore their applicability in biologically relevant conditions, such as crowded intracellular environments and systems with post-translational modifications.

## Methods

### Simulations of folded proteins.

All-atom MD simulations of Ubiquitin, GB3, BPTI, HEWL, Villin HP35 (with double nor-leucine substitutions at residues 24 and 29), protein complexes (Barnase/Barstar and SGPB/OMTKY3) were initiated in their folded states obtained from Protein Data Bank (PDB IDs: 1D3Z, 1P7E, 5PTI, 6LYZ, 2F4K, 1BRS, and 3SGB respectively). Systems were prepared in Gromacs 2021.5^78^ using truncated octahedron boxes with a length of 5.5 nm, except for HEWL and protein complexes, which required a larger box of 7.0 nm and 8.0 nm, respectively. After initial energy minimization in vacuum and solvation with TIP4P/2005 water,^22^ Na + and Cl − ions were added to achieve 150 mM salt concentration, using improved salt parameters from Lou and Roux.^79^ Systems were equilibrated in a canonical ensemble (NVT) ensemble using a Nose-Hoover thermostat^80^ with a coupling constant of 1.0 ps at 300 K, followed by an isothermal-isobaric ensemble (NPT) equilibration using a Berendsen barostat^81^ with an isotropic coupling constant of 5.0 ps at 1 bar. The Gromacs topologies and coordinates were then converted to Amber formats using ParmEd,^82^ with hydrogen mass repartitioning^83^ to 1.5 amu to enable a 4 fs timestep. Subsequent simulations were carried out using the Amber22 MD simulation package. The initial minimization was performed using the steepest descent and conjugate gradient algorithms, with non-hydrogen atoms restrained by a 5 kcal/mol/Å^2^ force constant. This was followed by two 5 ns NVT equilibration phases at 300 K: the first with reduced restraints (1 kcal/mol/Å^2^) and the second with all restraints removed. A subsequent 10 ns NPT equilibration employed a Monte Carlo barostat (with an isotropic coupling constant of 1.0 ps and a pressure of 1.0 bar) and Langevin dynamics for temperature regulation (with a friction coefficient of 1.0 ps^−1^). Bonds involving hydrogen atoms were constrained using the SHAKE algorithm.^84^ Short-range interactions were truncated at 0.9 nm, and long-range electrostatics were treated using the Particle Mesh Ewald (PME) method.^85,86^ Finally, four independent NPT production replicas were conducted for each protein, using the same parameters as those used for equilibration.

### Simulations of disordered proteins.

Conformational sampling of disordered proteins was performed using parallel tempering in the well-tempered ensemble (PT-WTE),^52,53^ a variant of temperature replica-exchange molecular dynamics (T-REMD).^87^ Simulations were conducted using Gromacs 2021.5 with the Plumed 2.8 plugin.^88,89^ For each disordered protein sequence (Table S1), an initial random coil conformation was generated and solvated in a truncated octahedron box containing 150 mM NaCl (using parameters from Lou and Roux) and counterions to neutralize the system. Fourteen temperature replicas, spanning 293–500 K, were employed. Long-range electrostatics were treated with the PME method with a 0.9 nm cutoff. To enhance computational efficiency, hydrogen mass repartitioning was employed, enabling a timestep of 5 fs. Bond constraints were applied using the LINCS algorithm.^90^ Following temperature equilibration, production runs were conducted in the NVT ensemble with biasing potentials (bias factor = 48) deposited every 4 ps. These potentials enhanced fluctuations in the system’s potential energy, resulting in a well-tempered ensemble and improved conformational exchange between replicas. The initial Gaussian potential height and width were 1.5 and 195.0 kJ/mol, respectively, with exchange probabilities between adjacent replicas ranging from 30–45%. The residue helix fraction for Httex1-Q16 was taken from Mohanty et al.,^91^ with an aggregate trajectory of ~ 45 μs.

Convergence of secondary structure propensities was assessed by analyzing the residual helicity at 302 K for each protein fragment. Consistent helix propensities were observed regardless of whether the entire trajectory, or trajectories excluding the initial 50 or 100 ns, were analyzed (Figure S11). Time-series plots of DSSP secondary structure assignments revealed transient structural behaviors, including periodic formation and melting of secondary structures throughout the trajectories (Figures S5, S6). These dynamic transitions facilitated convergence in estimated secondary structure propensities, underscoring the robustness of the PT-WTE approach for modeling disordered proteins.

### Trajectory analyses.

Structural properties were analyzed using Gromacs tools to calculate backbone root-mean-square deviations (RMSD), root-mean-square fluctuations (RMSF), and radius of gyration (R_g_). Small-angle X-ray scattering (SAXS) curves were generated using the CRYSOL,^92^ and R_g_ values were determined from these curves using Guinier analysis in ATLAS Primus.^93^ Chemical shift deviations were calculated using SPARTA +^66^ and compared to random coil reference values obtained from the Poulsen IDP server.^94^ Secondary structure assignments from simulations were determined using gmx do_dssp,^95^ which implements the DSSP algorithm,^67^ and compared to experimental propensities derived from NMR secondary chemical shifts using δ2d software.^68^ Solvent-accessible surface areas (SASA) were calculated using the gmx sasa utility, based on the algorithm of Eisenhaber et al.^96^ Backbone amide S^2^ order parameters for ubiquitin were computed using the iRED method^97,98^ within the Python package pyDR,^99,100^ with the trajectories divided into 1 μs blocks for analysis as recommended by Gu et al.^98^ Agreement between simulated and experimental observables was quantified by calculating the RMSD using ((x_sim_ – x_exp_)^2^/N)½, where N is the number of residues sampled, and x_sim_ and x_exp_ represent the simulated and experimental values, respectively. Three-dimensional structures and molecular dynamics trajectories were visualized and animated using the VMD software.^101^

The relevant force fields can be downloaded at https://bitbucket.org/jeetain/all-atom_ff_refinements.
